# Carbolithiation of *N*-alkenyl ureas and *N*-alkenyl carbamates

**DOI:** 10.3762/bjoc.9.70

**Published:** 2013-03-28

**Authors:** Julien Lefranc, Alberto Minassi, Jonathan Clayden

**Affiliations:** 1 School of Chemistry, University of Manchester, Oxford Road, Manchester M13 9PL, UK

**Keywords:** carbamate, carbolithiation, carbometallation, organolithium, stereospecificity, styrene, urea

## Abstract

*N*-Alkenyl ureas and *N*-alkenyl carbamates, like other *N*-acyl enamines, are typically nucleophilic at their β-carbon. However, by incorporating an α-aryl substituent, we show that they will also undergo attack at the β-carbon by organolithium nucleophiles, leading to the products of carbolithiation. The carbolithiation of *E* and *Z N*-alkenyl ureas is diastereospecific, and *N*-*tert*-butoxycarbonyl *N*-alkenyl carbamates give carbolithiation products that may be deprotected in situ to provide a new connective route to hindered amines.

## Introduction

Enamines and *N*-acyl enamines are in general nucleophiles, reacting with electrophiles at the carbon atom β to the nitrogen atom [1–2]. The resulting intermediate iminium or *N*-acyliminium ions are electrophilic, and may themselves trap a nucleophile at the position α to the nitrogen substituent. However, we [3–4] and others [5–7] have shown that this typical reactive polarity may be reversed when *N*-acylenamines (especially *N*-vinyl ureas [8]) meet organolithium nucleophiles. *N*-Carbamoyl enamines bearing α-aryl substituents (in other words, α-acylaminostyrenes), may undergo reaction as electrophiles, with the carbon atom β to nitrogen succumbing to attack by organolithium nucleophiles in an enamine carbolithiation reaction [9]. Similar reactivity is observed with related *O*-carbamoyl enols [10–12]. The organolithium resulting from the enamine carbolithiation is nucleophilic at the atom α to nitrogen, and such carbolithiations have been used to generate hindered organolithiums as intermediates for further rearrangement reactions [13], for example intramolecular acylation [6], arylation [3–4] or vinylation [4]. In this paper, we now report our studies on the scope of the carbolithiation–protonation of styrenes carrying α-acylamino substituents, namely *N*-alkenyl ureas and *N*-alkenyl carbamates.

## Results and Discussion

**Scheme 1:**
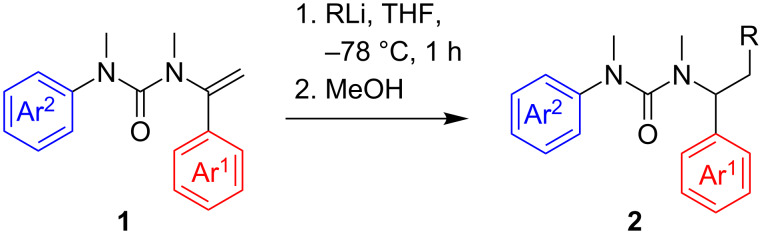
Carbolithiation of ureas **1**.

Simple *N*-alkenyl ureas **1** were prepared by a reported method [2] entailing N-acylation of an acetophenimine with an isocyanate, followed by N-alkylation of the resulting urea. When urea **1a** was treated with *t*-BuLi or *s*-BuLi in THF at −78 °C for one hour, followed by protonation, carbolithiated products **2a** and **2b** were isolated in good yield (Scheme 1 and Table 1, entries 1 and 2). Similar reactivity was observed between urea **1a** and less hindered organolithiums such as iPrLi or *n*-BuLi [3], but in THF even at −78 °C a rearrangement [14–18] of the intermediate benzyllithium reduces the yield of the simple carbolithiation product. However, by lowering the temperature to −85 °C rearrangement occured to only a limited extent, and the addition product **2c** was obtained in 53% yield (Table 1, entry 3). Rearrangement was also suppressed if the substituent Ar^2^ was replaced by either a *p*-chlorophenyl or a *p*-methoxyphenyl ring, and even with *n*-BuLi the carbolithiation–protonation product may be obtained in moderate yield from **1b** and **1c** (Table 1, entries 4 and 5).

**Table 1:** Organolithium addition to ureas **1**.

Entry	SM	Ar^1^	Ar^2^	R	**2**, yield (%)

1	**1a**	Ph	Ph	*t*-Bu	**2a**, 77^a^
2	**1a**	Ph	Ph	*s*-Bu	**2b**, 74^b^
3	**1a**	Ph	Ph	iPr	**2c**, 53^c^
4	**1b**	Ph	4-MeOC_6_H_4_	*n*-Bu	**2d**, 61
5	**1c**	Ph	4-ClC_6_H_4_	*n*-Bu	**2e**, 47

^a^Reported in ref [3]; ^b^mixture of diastereoisomers; ^c^reaction carried out at −85°C.

**Scheme 2:**
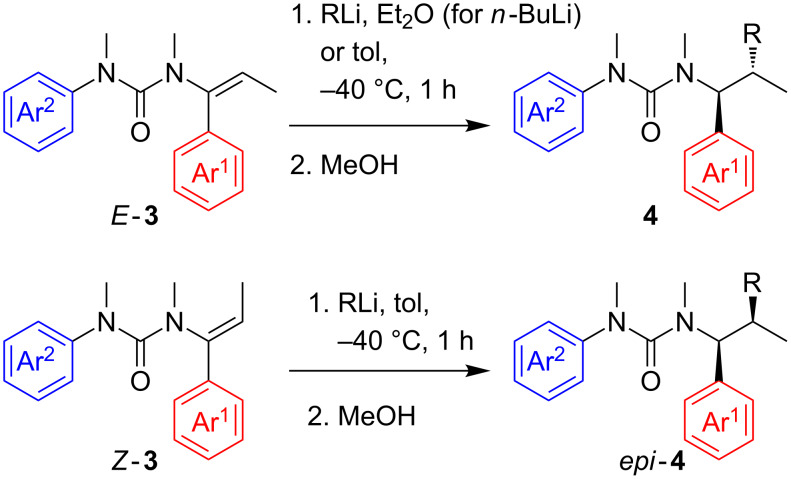
Diastereospecific carbolithiation of ureas **3**.

β-Substituted vinyl ureas **3** are available as either *E* or *Z* geometrical isomers according to the method of synthesis: N-acylation of a propiophenimine typically generates an *E*-alkenylurea, but deprotonation and reprotonation of the urea inverts its geometry to *Z* [2], probably via an intramolecularly chelated urea-substituted allyl anion [17]. Urea *E*-**3a** was treated with *n*-BuLi in Et_2_O (the less-coordinating solvent suppresses rearrangement of the product [4]) at −40 °C: it underwent clean carbolithiation of the double bond, and on protonation urea **4a** was obtained as a single diastereomer in 85% yield (Scheme 2 and Table 2, entry 1). Other *E*-alkenyl ureas bearing a range of substituted aromatic rings *E*-**3b**–**f** were likewise treated with alkyllithium reagents *s*-BuLi, iPrLi and *t*-BuLi, this time in toluene. As before, a noncoordinating solvent was used to suppress rearrangement. In each case the addition product **4b**–**f** was obtained in good yield always as a single diastereomer (Table 2, entries 2–5 and 7). Styrenes with substituents on the aromatic ring underwent carbolithiation irrespective of the electronic character of the ring: with electron-rich or electron-poor aromatic rings Ar^1^ carbolithiation was complete in one hour.

**Table 2:** Organolithium additions to ureas **3**.

Entry	SM	Ar^1^	Ar^2^	R	**4**, yield (%)

1	*E*-**3a**	Ph	3-MeOC_6_H_4_	*n*-Bu	**4a**, 85
2	*E*-**3b**	4-F-C_6_H_4_	Ph	*s*-Bu	**4b**, 70^a^
3	*E*-**3c**	Ph	4-MeC_6_H_4_	iPr	**4c**, 78
4	*E*-**3d**	4-MeC_6_H_4_	Ph	*t*-Bu	**4d**, 63
5	*E*-**3e**	4-ClC_6_H_4_	Ph	iPr	**4e**, 81
6	*Z*-**3e**	4-ClC_6_H_4_	Ph	iPr	*epi***-4e**, 80
7	*E*-**3f**	Ph	4-MeOC_6_H_4_	*n*-Bu	**4f**, 85
8	*E*-**3f**	Ph	4-MeOC_6_H_4_	iPr	**4g**, 85^b^
9	*Z*-**3f**	Ph	4-MeOC_6_H_4_	iPr	*epi***-4g**, 85^b^
10	*E-***3g**	4-ClC_6_H_4_	4-MeOC_6_H_4_	iPr	**4h**, 60

^a^2:1 mixture of diastereoisomers; ^b^reaction reported in ref [3] but yield now improved.

When the reaction was performed using the *Z*-isomer of the starting materials, *Z*-**3e** and *Z*-**3f** (Scheme 2 and Table 2, entries 6 and 8), the other diastereomer of the product urea *epi*-**4** was obtained selectively: the carbolithiation–protonation is completely diastereospecific. Both geometrical isomers of **3** presented similar reactivity and the products **4** were obtained in similar yields under the same reaction conditions.

**Scheme 3:**
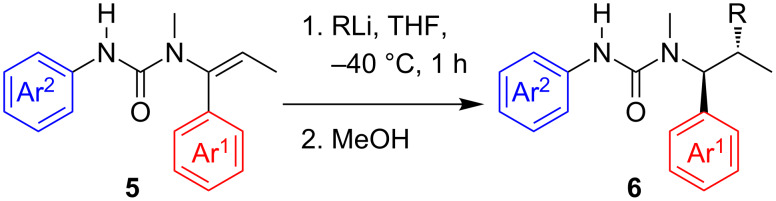
Diastereospecific carbolithiation of ureas **5**.

To avoid possible contamination of the carbolithiation products by compounds arising from tandem carbolithiation–rearrangement, we were also keen to explore the possibility of carbolithiating vinyl ureas incapable of rearrangement, either because they lack the *N*’-aryl substituent or because the remote nitrogen is protected from attack by existing as an anion. Urea **5a**, which was available as an intermediate from the synthesis of **3a**, was treated with *n*-BuLi, using an additional equivalent of the organolithium to allow for deprotonation of the urea NH (Scheme 3) and in THF since a competing rearrangement is not a problem. Despite the carbolithiation now requiring an anion to act as an electrophile, the corresponding carbolithiated and protonated product **6a** was obtained as a single diastereoisomer in excellent yield without chromatography (Table 3, entry 1) after one hour in THF at −40 °C. With urea **5b** primary (*n*-BuLi), secondary (iPrLi) and also tertiary (*t*-BuLi) alkyllithium reagents were added successfully in excellent yields (Table 3, entries 2–4), and no chromatography was needed.

**Table 3:** Carbolithiation of urea **5**.

Entry	SM	Ar^1^	Ar^2^	R	**6**, yield (%)

1	**5a**	Ph	4-MeOC_6_H_4_	*n*-Bu	**6a**, 80
2	**5b**	4-ClC_6_H_4_	4-MeOC_6_H_4_	*n*-Bu	**6b**, 87
3	**5b**	4-ClC_6_H_4_	4-MeOC_6_H_4_	iPr	**6c**, 98
4	**5b**	4-ClC_6_H_4_	4-MeOC_6_H_4_	*t*-Bu	**6d**, 98

**Figure 1:**
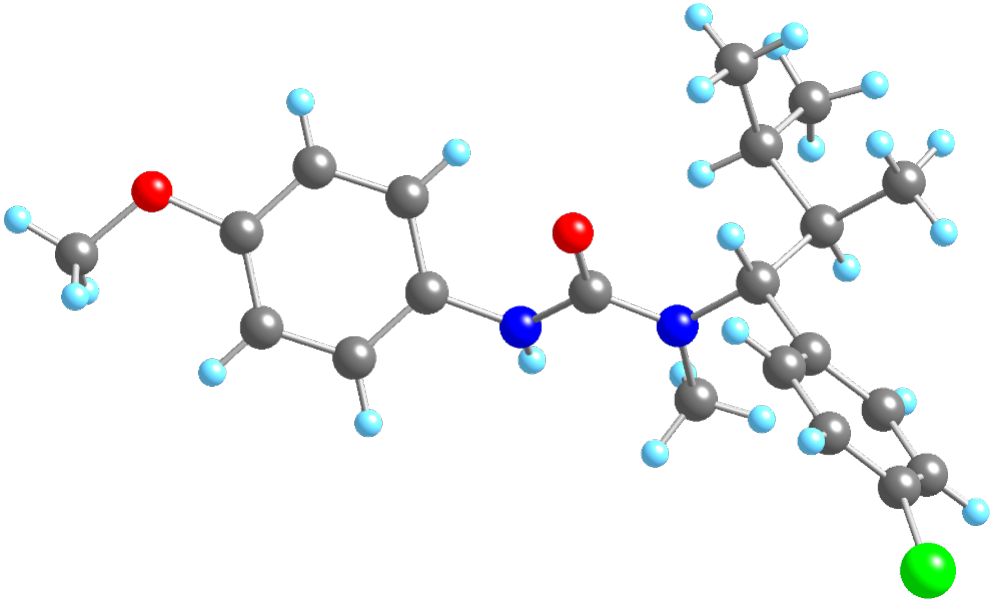
X-ray crystal structure of urea **6c**.

The relative configuration of the carbolithiation products **6** was established by X-ray crystallography of urea **6c** (Figure 1). The stereochemical outcome of the reaction is consistent with *syn*-addition of the organolithium across the double bond (as is typical for carbolithiation [9,19]) followed by retentive protonation.

The relative configuration of the carbolithiation products **4** was likewise confirmed by methylation (NaH, MeI) of **6d** to provide a single diastereoisomer of urea **4g** in 60% yield, which was spectroscopically identical with the compound obtained by treating urea *E*-**3g** with iPrLi (Table 2, entry 9). Again, the stereochemical outcome is consistent with *syn*-carbolithiation followed by retentive protonation.

**Scheme 4:**
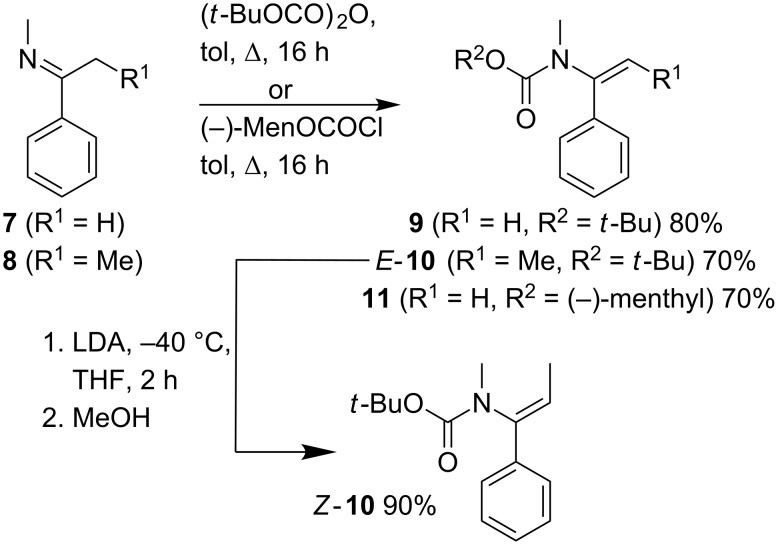
Synthesis of *N*-alkenyl carbamates **9**–**11**.

**Figure 2:**
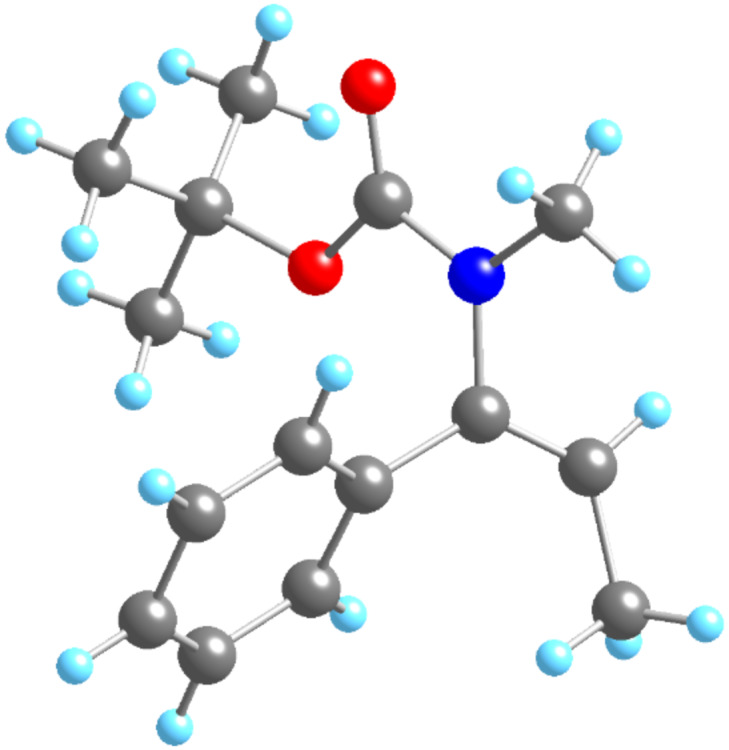
X-ray crystal structure of carbamate *E*-**10**.

In principle, the urea products **2**, **4** and **6** could be solvolysed to liberate free amines, as has been demonstrated for related compounds [4,15,20]. However, we reasoned that the related *tert*-butyloxycarbonyl-substituted carbamates would give more readily manipulated carbamate products bearing a standard Boc protecting group, providing they too could be carbolithiated and trapped without rearrangement. Related carbamates are reactive towards carbolithiation–rearrangement reactions [6]. Thus, Boc-protected carbamates **9**–**11** were synthesised by acylation of the imines **7** and **8** with di-*tert*-butyl dicarbonate or with (−)-menthylchloroformate (Scheme 4). The *N*-alkenylcarbamates **10** and **11** were formed exclusively as their *E* isomers, and the X-ray crystal structure of *E*-**10** is shown in Figure 2.

**Scheme 5:**
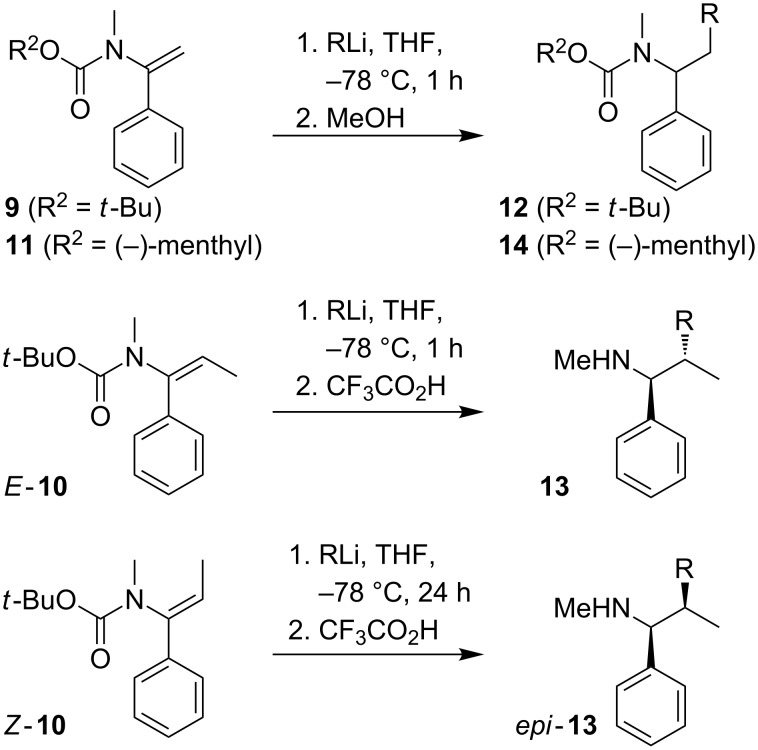
Umpolung carbolithiation of carbamates **9** and **10**.

Vinyl carbamate **9** was treated with primary, secondary or tertiary alkyllithium reagents at −78 °C in THF for one hour (Scheme 5), and after protonation the addition products **12a**–**c** were isolated in good yields (Table 4, entries 1–3) . Substituted carbamate **10** also reacted with primary, secondary or tertiary alkyllithium reagents under similar conditions, and in this case the carbamates were deprotected by treatment with CF_3_CO_2_H in a one-pot process, to provide the amines **13a**–**c** in good yields over the two steps (Table 4, entries 4–6). In every case, the amine **13** was obtained as a single diastereomer, which we assume, by analogy with the reactions of the equivalent ureas, to be that shown, arising from *syn*-carbolithiation and retentive protonation.

**Table 4:** Organolithium addition to vinyl carbamates.

Entry	SM	R	Product, yield (%), (dr)

1	**9**	*n*-Bu	**12a**, 61
2	**9**	iPr	**12b**, 61
3	**9**	*t*-Bu	**12c**, 80
4	*E*-**10**	*n*-Bu	**13a**, 70 (>95:5)
5	*E*-**10**	iPr	**13b,** 80 (>95:5)
6	*E*-**10**	*t*-Bu	**13c,** 81 (>95:5)
7^a^	*Z-***10**	iPr	*epi*-**13b,** 50 (80:20)
8	**11**	iPr	**14**, 60 (50:50)

^a^24 h reaction time.

*E*-**10** was isomerised to *Z*-**10** by treatment with LDA and reprotonation (presumably, like the equivalent ureas [2–3], via an intramolecularly coordinated *Z*-allyllithium [21–22]), giving *Z*-**10** in excellent yield (Scheme 4). However, in contrast to *Z*-alkenyl ureas, *Z*-**10** was rather less reactive than its *E*-isomer. The carbolithiation with iPrLi (Scheme 5) was slower, and had to be performed for 24 hours instead of 1 hour. After deprotection with trifluoroacetic acid, the amine *epi*-**13b** was obtained in lower yield (50%) and as a 8:2 mixture of diastereomers (Table 4, entry 7). The loss of diastereospecificity may be explained by the long reaction time: we assume that *syn*-carbolithiation is followed by a partial epimerisation of the intermediate organolithium during the 24 h before the reaction is quenched.

Related vinylureas will undergo enantioselective carbolithiation in the presence of (−)-sparteine or a (+)-sparteine surrogate [4], but enantioselective carbolithiation of carbamate **9** in the presence of (−)-sparteine led to product with only 60:40 er. The use of a chiral auxiliary in the form of a (−)-menthylcarbamate (**11**) also failed to induce selectivity, reacting with iPrLi to yield a carbolithiated product **14** in 60% yield as a 50:50 mixture of diastereoisomers (Table 4, entry 8).

## Conclusion

In conclusion, we have demonstrated that electron-rich double bonds of vinyl ureas and carbamates may undergo carbolithiation with primary, secondary and tertiary organolithium reagents. *N*-*tert*-butoxycarbonyl vinylcarbamates may be carbolithiated, protonated and deprotected in a one-pot synthesis of amines employing this unusual umpolung nucleophilic β-alkylation. With β-substituted vinylureas, the carbolithiation is diastereospecific, with (*E*) and (*Z*)-isomers of the ureas giving different diastereoisomers of the products; (*E*)-*N*-alkenylcarbamates react with complete diastereospecificity. The overall *syn*-relative configuration of the reaction products, which probably arises from *syn*-carbolithiation followed by retentive protonation, was confirmed by X-ray crystallography.

## Experimental

1-(4-Methoxyphenyl)-1,3-dimethyl-3-[(1R*,2R*)-2-methyl-1-phenylhexyl]urea (4f): To a solution of urea **3f** (0.086 g, 0.28 mmol, 1 equiv) in dry toluene (0.1 M) cooled to −40 °C, *n*-BuLi (2 equiv) was added slowly. After 1 h at −40 °C, the reaction was quenched slowly with MeOH and a saturated aqueous solution of NH_4_Cl. The resulting solution was extracted with EtOAc, dried with MgSO_4_, concentrated under reduced pressure and purified by flash chromatography on silica gel (eluting with petroleum ether/EtOAc 9:1). The title compound **4f** (0.086g, 85%) was obtained as a colourless oil. *R*_f_ 0.5 (PE/EtOAc 8:2); IR (film) ν_max_ (cm^−1^): 2957, 2931, 1651, 1644, 1510; ^1^H NMR (400 MHz, CDCl_3_) δ 7.30–7.17 (m, 5H, 5 × Ar*H*), 6.79 (dt, *J* = 8.8, 2.5 Hz, 2H, 2 × Ar*H*), 6.66 (dt, *J* = 8.8, 2.5 Hz, 2H, 2 × Ar*H*), 5.02 (d, *J* = 12.0 Hz, 1H, C*H*-N), 3.70 (s, 3H, O-C*H_3_*), 3.05 (s, 3H, N-C*H_3_*), 2.21 (s, 3H, N-C*H_3_*), 2.04 (m, 1H, C*H*-CH_3_), 1.20 (m, 6H, 3 × C*H_2_*), 0.88 (t, *J* = 7.6 Hz, 3H, C*H_3_*-CH_2_), 0.70 (d, *J* = 6.4 Hz, 3H, C*H_3_*-CH); ^13^C NMR (100 MHz, CDCl_3_) δ 162.6 (C=O), 156.6 (*C*_ar_-OCH_3_), 140.1 (C_ar_), 139.2 (C_ar_), 128.6 (2 × CH_ar_), 128.1 (2 × CH_ar_), 127.1 (CH_ar_), 126.1 (2 × CH_ar_), 114.4 (2 × CH_ar_), 64.3 (CH-N), 55.3 (O-CH_3_), 41.0 (N-CH_3_), 32.6 (*C*H-CH_3_), 32.1 (*C*H_2_-CH), 30.7 (N-CH_3_), 29.2 (*C*H_2_-CH_2_-CH_3_), 23.1 (*C*H_2_-CH_3_), 17.1 (*C*H_3_-CH), 14.2 (*C*H_3_-CH_2_); HRMS–ES (*m*/*z*): [M + H]^+^ calcd for C_23_H_33_N_2_O_2_, 369.2537; found, 369.2536.

## Supporting Information

File 1 Experimental procedures for the synthesis of all new compounds.

File 2 cif file for **6c**.

File 3 cif file for *E*-**10**.
