# Supplementary material for: Carbolithiation of N-alkenyl ureas and N-alkenyl carbamates
Source: Beilstein J Org Chem. 2013 Mar 28;9:628–32. doi: 10.3762/bjoc.9.70 (PMC3628850; doi:10.3762/bjoc.9.70)
Supplement: File 1 — Experimental procedures for the synthesis of all new compounds. [file Beilstein_J_Org_Chem-09-628-s001.pdf]

Supporting Information

for

Carbolithiation of *N*-alkenyl ureas and *N*-alkenyl carbamates

Julien Lefranc, Alberto Minassi and Jonathan Clayden*

School of Chemistry, University of Manchester, Oxford Road, Manchester M13 9PL, UK

Email: Jonathan Clayden - clayden@man.ac.uk

* Corresponding author

Experimental procedures for the synthesis of all new compounds

Contents

General Information S2
General procedures S3
Experimental procedures and characterisation data S4–S17
References S17

General Information

NMR spectra were recorded on a Bruker Ultrashield 300, 400 or 500 MHz spectrometer. The chemical shifts (δ) are reported in ppm downfield of trimethylsilane, and coupling constants (*J*) are reported in Hertz and rounded to 0.1 Hz. Splitting patterns are abbreviated as follows: singlet (s), doublet (d), triplet (t), quartet (q), septet (sep), multiplet (m), broad (br), or a combination of these. Solvents were used as internal standard when assigning NMR spectra (δ H: $CDCl_3$ 7.26 ppm, MeOD 3.31 ppm; δ C: $CDCl_3$ 77.0 ppm, MeOD 49.0 ppm).
Low- and high-resolution mass spectra were recorded by staff at the University of Manchester. EI and CI spectra were recorded on a Micromass Trio 2000; ES and APCI spectra were recorded on a Micromass Platform II; high-resolution mass spectra (HRMS, EI and ES) were recorded on a Thermo Finnigan MAT95XP mass spectrometer. Infrared spectra were recorded on a Perkin Elmer Spectrum RX I FTIR spectrometer as a film on a sodium chloride plate. Absorptions reported are sharp and strong; only absorption maxima of interest are reported. Melting points (mp) were determined on a Gallenkamp apparatus and are uncorrected.
Thin-layer chromatography (TLC) was performed using commercially available precoated plates (Macherey-Nagel alugram Sil G/UV254) and visualised with UV light at 254 nm or phosphomolybdic acid dip (5% in ethanol). Flash chromatography was carried out using Fluorochem Davisil 40–63u 60 Å.
All reactions were conducted under an atmosphere of dry nitrogen or argon in oven-dried glassware. Tetrahydrofuran (THF) and diethylether (Et_2O) were distilled under nitrogen from sodium using benzophenone as indicator. Dichloromethane and toluene were obtained by distillation from calcium hydride under nitrogen. Petrol refers to the fraction of light petroleum ether boiling between 40–65 °C. All other solvents and commercially obtained reagents were used as received or purified using standard procedures.
The organolithium reagents were used directly from commercially available solutions from Sigma-Aldrich: *n*-BuLi (2.5 M in hexanes), *s*-BuLi (1.4 M in cyclohexane), *t*-BuLi (1.7 M in pentane) and iPrLi (0.7 M in pentane).

General procedures

General procedure 1: Carbolithiation of dimethylated ureas **3**.

To a solution of urea **3** in dry toluene (0.1 M) cooled at −40 °C, the desired organolithium reagent (2 equiv) is added slowly. After 1 hour, the reaction is quenched slowly with MeOH and NH_4Cl. The crude is extracted with EtOAc, dried with $MgSO_4$, concentrated under reduced pressure and purified by chromatography on silica gel (PE/EtOAc 9:1).

General procedure 2: Carbolithiation of monomethylated ureas **5**.

To a solution of urea **5** in dry tetrahydrofuran (0.1 M) cooled at −40 °C, the desired organolithium reagent (3 equiv) is added slowly. After 1 hour, the reaction is quenched slowly with MeOH and NH_4Cl. The crude is extracted with EtOAc, dried with $MgSO_4$, and concentrated under reduced pressure. The product was obtained without further purification.

General procedure 3: Carbolithiation of *gem* disubstituted ene-carbamates **9**.

To a solution of carbamate **9** in dry THF (0.1 M), cooled at −78 °C, the desired organolithium reagent (2 equiv) is added slowly. After 1 hour at −78 °C, the reaction is quenched by slow addition of MeOH. The mixture is extracted with EtOAc and washed with NH_4Cl. The organic phase is dried ($MgSO_4$), filtered and concentrated under reduced pressure. The crude is purified by flash chromatography on silica gel (PE/DCM 1:1).

General procedure 4: Carbolithiation of trisubstituted ene-carbamates **10** followed by deprotection.

Carbamate **10** is solubilised in dry THF (0.1 M) and the mixture is cooled to −40 °C. The desired alkyllithium reagent is added slowly and the reaction is stirred for 1 hour at −40 °C. The reaction is then quenched by slow addition of methanol. The solvent is removed under reduced pressure and the crude is solubilised in TFA. The reaction is stirred for 1 hour at r.t. The reaction mixture is diluted in DCM and washed with $NaHCO_3$ (1 M). The organic phase is dried ($MgSO_4$) and filtered, and the solvent is removed under reduce pressure. The corresponding amine is obtained without further purification.

Experimental procedures and characterisation data

Synthesis of the starting materials:

Ureas **1a**, **1b**, **1c**, **3a**, **3b**, **3c**, **3d**, **3e**, **3f**, **3g** and **5b** were synthesised according to the procedures described [1,2].

Compounds **2a** and **4a** were previously reported [2]

1,3-Dimethyl-1-(3-methyl-1-phenylbutyl)-3-phenylurea (2c)

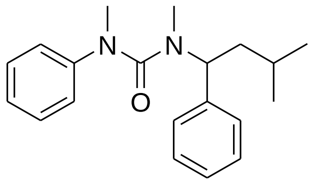

To a stirred solution of urea **1a** (100 mg; 0.376 mmol) in dry THF (2.5 mL) at −85 °C, iPrLi (1.076 mL; 0.753 mmol; 2 equiv) was added resulting in a red solution. The reaction was stirred at −85 °C for 1 h then quenched with MeOH and NH_4Cl (sat. sol). The crude was extracted with EtOAc, dried with $MgSO_4$, concentrated under reduced pressure and purified by chromatography on silica gel (petrol/EtOAc 98:2) to afford the desired carbolithiated product **2c** (62 mg, 53%) as a colourless oil.

IR ν_{max} ($CHCl_3$)/cm^{-1}: 3060, 3029, 2955, 2867, 1644, 1595, 1494, 1454, 1366, 1337.

^{1}H NMR (400 MHz, $CDCl_3$): δ 7.23-7.16 (m, 7 H), 7.02-6.94 (m, 3H), 5.43 (t, *J* = 8.4 Hz, 1H), 3.14 (s, 3H), 2.19 (s, 3H), 1.64 (m, 2H), 1.42 (m, 1H), 0.92 (d, *J* = 6.4 Hz, 3H), 0.82 (d, *J* = 6.8 Hz, 3H).

^{13}C NMR ($CDCl_3$, 100 MHz): δ 162.3, 147.1, 140.1, 129.3, 128.2, 127.9, 127.1, 124.5,124.3, 56.4, 40.35, 39.4, 30.86, 24.95, 22.8, 22.5.

HRMS–ESI: calcd for $C_{20}H_{27}N_2O$ ($M+H^+$) 311.2118, found 311.2117.

1-(4-Methoxyphenyl)-1,3-dimethyl-3-(1-phenylhexyl)urea (2d)

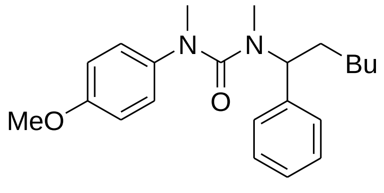

To a stirred solution of urea **1b** (50 mg; 0.168 mmol) in dry THF (2.5 mL) at −78 °C, *n*-BuLi (135 μL; 0.336 mmol; 2 equiv) was added resulting in a red solution. The reaction was stirred at −78 °C for 1 h then quenched with MeOH and NH_4Cl (sat. sol). The crude was extracted with a mixture 3:1 petrol/Et_2O, dried with $MgSO_4$, concentrated under reduce pressure and purified by chromatography on silica gel (petrol/EtOAc 98:2) to afford the desired carbolithiated product **2d** (36 mg, 61%) as a colourless oil.

IR ν_{max} ($CHCl_3$)/cm^{-1}: 2930, 2859, 1633, 1510, 1454, 1385, 1335.

^{1}H NMR (500 MHz, $CDCl_3$): δ 7.30-7.20 (m, 5H), 6.99-6.97 (m, 2H), 6.79-6.78 (m, 2H), 5.35 (t, *J* = 7.7 Hz, 1H), 3.76 (s, 3H), 3.14 (s, 3H), 2.23 (s, 3H), 1.87-1.82 (m, 1H), 1.73-1.67 (m, 1H), 1.34-1.18 (m, 6H), 0.88 (t, *J* = 6.8 Hz, 3H).

^{13}C NMR ($CDCl_3$, 100 MHz): δ 162.7, 156.8, 140.4, 140.2, 128.2, 127.8, 127.0, 126.4, 114.5, 58.3, 55.4, 41.0, 31.8, 30.7, 30.4, 26.3, 22.6, 14.0.

HRMS–ESI: calcd for $C_{22}H_{30}N_2O_2Na$ ($M+Na^+$) 377.2199, found 377.2195.

1-(4-Chlorophenyl)-1,3-dimethyl-3-(1-phenylhexyl)urea (2e)

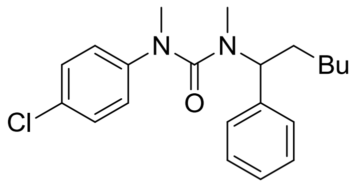

To a stirred solution of urea **1c** (50 mg; 0.166 mmol) in dry Et_2O (2.5 mL) at −78 °C, *n*-BuLi (133 µL; 0.332 mmol; 2 equiv) was added resulting in a red solution. The reaction was stirred at −78 °C for 1 h then quenched with MeOH and NH_4Cl (sat. sol). The crude was extracted with a mixture 3:1 petrol/Et_2O, dried with $MgSO_4$, concentrated under reduce pressure and purified by chromatography on silica gel (petrol/EtOAc 98:2) to afford the desired carbolithiated product **2e** (28 mg, 47%) as a colourless oil.

IR ν_{max} ($CHCl_3$)/cm^{-1}: 2954, 2929, 1651, 1493, 1385, 1330.

^{1}H NMR (400 MHz, $CDCl_3$): δ 7.35-7.20 (m, 7H), 6.98-6.94 (m, 2H), 5.39 (t, *J* = 7.8 Hz, 1H), 3.19 (s, 3H), 2.30 (s, 3H), 1.95-1.86 (m, 1H), 1.81-1.72 (m, 1H), 1.36-1.25 (m, 6H), 0.92-0.89 (m, 3H).

^{13}C NMR ($CDCl_3$, 100 MHz): δ 162.0, 145.6, 139.9, 129.6, 129.4, 128.3, 127.8, 127.3, 125.2, 58.5, 40.1, 31.7, 30.7, 30.3, 26.4, 22.6, 14.0.

HRMS–ESI: calcd for $C_{21}H_{27}N_2OClNa$ (M+Na^+) 381.1704, found 381.1700.

1-[(1*R,2*R**)-1-(4-Fluorophenyl)-2,3-dimethylpentyl]-1,3-dimethyl-3-phenylurea (4b)**

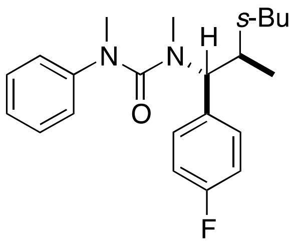

The compound was synthesised following the general procedure 1 starting from 113 mg (0.38 mmol) of urea ***E*-3b**. The desired product was obtained in 70% yield (95 mg) as a colourless oil. (Mixture of diastereomers 2:1)

R

R_f: 0.5 (PE/EtOAc 8:2).

IR ν_{max} (film)/cm^{-1}: 3062, 3038, 2963, 2929, 2876, 2242, 1651 and 1644.

^{1}H NMR (300 MHz, $CDCl_3$): δ 7.43-6.90 (m, 18H, 9xAr*H*), 5.32 (d, *J* = 12.0 Hz, 1H, C*H*-N major), 5.31 (d, *J* = 12.0 Hz, 1H, C*H*-N minor), 3.18 (s, 3H, N-C*H₃*, major) and 3.17 (s, 3H, N-CH_3, minor), 2.33 (s, 3H, N-CH_3 minor), 2.28 (s, 3H, N-CH_3 major), 2.18 (m, 2H, C*H*-CH_3), 1.77-1.69 (m, 1H, CH_2-CH_3 minor) 1.49-1.28 (m, 4H, CH_3-C*H*-CH_2-CH_3 major and CH_3-C*H*-CH_2 minor), 1.04-0.92 (m, 14H, 2xCH_3 both and CH_2-CH_3 minor), 0.62 (d, *J* = 6.6 Hz, 3H, CH_3-CH minor) and 0.61 (d, *J* = 6.6 Hz, 3H, CH_3-CH major).

^{13}C NMR (75 MHz, $CDCl_3$): δ 162.1 (C=O major), 162.0 (C=O minor), 161.9 (d, J_F = 243.7 Hz, C_{ar}-F major), 161.8 (d, J_F = 243.9 Hz, C_{ar}-F minor), 147.1 (C_{ar} major), 147.0 (C_{ar} minor), 135.1 (J_F = 3.5 Hz, C_{ar}), 130.4 (d, J_F = 7.7 Hz, 2xCH_{ar} major), 130.2 (d, J_F = 8.2Hz 2xCH_{ar} minor), 129.3 (2xCH_{ar} minor), 129.2 (2xCH_{ar} major), 124.6 (2xCHar minor), 124.5 (2xCH_{ar} major), 124.4 (CH_{ar} minor), 124.0 (CH_{ar} major) 114.9 (d, J_F = 20.9 Hz 2xCH_{ar} major), 114.9 (d, J_F = 21.7 Hz 2xCH_{ar} minor), 61.0 (CH-N minor), 60.8 (CH-N major), 40.5 (N-CH_3 major), 40.4 (N-CH_3 minor), 38.2 (*C*H-CH_3 minor), 35.3 (*C*H-CH_3 major), 33.9 (CH_3-*C*H-CH_2 major), 33.6 (CH_3-*C*H-CH_2 minor), 30.6 (N-CH_3 minor), 30.5 (N-CH_3 major), 28.5 (*C*H_2-CH_3 major), 21.9 (CH_2 minor), 18.1 (CH_3 major), 13.0 (CH_3 major), 12.5 (CH_3 minor), 12.5 (CH_3 minor), 11.2 (CH_3 minor) and 10.7 (CH_3 major).

HRMS–ES: 357.2337 for $C_{22}H_{29}N_2OF$ (M+H^+) found 357.2340.

1-((1*R,2*R**)-2,3-Dimethyl-1-phenylbutyl)-1,3-dimethyl-3-(*p*-tolyl)urea (4c)**

The compound was synthesised following the general procedure 1 starting from urea ***E*-3c**. The desired product was obtained in 78% yield as a colourless oil.

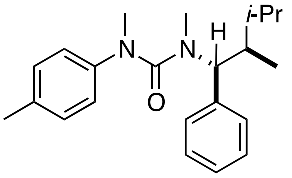

***R*f**: 0.5 (PE/EtOAc 8:2).

IR ν_{max} (film)/cm^{-1}: 2961, 2926, 2873,2242, 1890, 1645 1582 and 1514.

^{1}H NMR (400 MHz; $CDCl_3$): δ 7.36-7.25 (m, 5H, 5xAr*H*), 6.97 (d, *J* = 8.5 Hz, 2H, 2xAr*H*), 6.74 (d, *J* = 8.4 Hz, 2H, 2xAr*H*), 5.25 (d, *J* = 12.2 Hz, 1H, C*H*-N), 3.11 (s, 3H, N-C*H₃*), 2.28 (s, 3H, C_{Ar}-CH_3), 2.27 (s, 3H, N-CH_3), 2.11 (dqd, *J* = 12.2, 6.6, 2.5 Hz, 1H, C*H*-CH_3), 1.77 (dtd, *J* = 13.8, 6.9, 2.5 Hz, 1H, C*H*(CH_3)$_2$), 0.96 (d, *J* = 6.9 Hz, 6H, 2x(CH_3)$_2$CH), 0.61 (d, *J* = 6.6 Hz, 3H, CH_3-CH).

^{13}C NMR (100 MHz; $CDCl_3$): δ 162.3 (C=O), 144.6 (C_{ar}), 139.2 (C_{ar}), 134.2 (C_{ar}), 129.8 (2xCH_{ar}), 128.8 (2xCH_{ar}), 128.1 (2xCH_{ar}), 127.1 (CH_{ar}), 124.3 (2xCH_{ar}), 62.0 (CH-N), 40.7 (N-CH_3), 36.9 (*C*H-CH_3), 30.7 (N-CH_3), 26.6 (*C*H-(CH_3)$_2$), 21.8 (CH-(*C*H_3)$_2$), 20.8 (C_{ar}-*C*H_3), 15.2 (CH-(*C*H_3)$_2$), 10.5 (*C*H_3-CH).

HRMS–ES: calcd for $C_{22}H_{30}N_2O$ 361.2250 found 361.2253 $[M + Na]^+$.

1,3-Dimethyl-1-phenyl-3-[(1*R,2*R**)-2,3,3-trimethyl-1-(*p*-tolyl)butyl]urea (4d)**

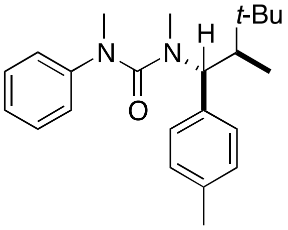

The compound was synthesised following the general procedure 1 starting from 80 mg (0.27 mmol) of urea ***E*-3d**. The desired product was obtained in 63% yield (60 mg) as a colourless oil.

***R*f**: 0.5 (PE/EtOAc 8:2).

IR ν_{max} (film)/cm^{-1}: 2965, 1642 and 1596.

^{1}H NMR (300 MHz, $CDCl_3$): δ 7.09 (s, 4H, 4xAr*H*), 7.03-6.93 (m, 3H, 3xAr*H*), 6.64-6.60 (m, 2H, 2xAr*H*), 5.43 (d, *J* = 9.6 Hz, 1H, C*H*-N), 3.12 (s, 3H, N-CH_3), 2.35 (s, 3H, C_{ar}-CH_3), 2.29 (s, 3H, N-CH_3), 2.09 (dq, *J* = 9.6 and 6.9 Hz, 1H, C*H*-CH_3), 1.04 (s, 9H, (CH_3)$_3$C) and 0.67 (d, *J* = 6.9 Hz, 3H, CH_3-CH).

^{13}C NMR (75 MHz, $CDCl_3$): δ 161.4 (C=O), 146.6 (C_{ar}), 137.1 (C_{ar}), 136.2 (*C*ar-CH_3), 129.0 (2xCH_{ar}), 128.7 (2xCH_{ar}), 128.3 (2xCH_{ar}), 123.9 (CH_{ar}), 123.5 (2xCH_{ar}), 62.3 (CH-N), 40.5 (*C*H-CH_3), 40.2 (N-CH_3), 33.9 (*C*-(CH_3)$_3$), 32.6 (N-CH_3), 28.2 (3x(*C*H_3)-C), 21.0 (C_{ar}-*C*H_3) and 12.9 (*C*H_3-CH).

HRMS–ES: calcd for $C_{23}H_{32}N_2ONa$ 375.2407 found 375.2410 $[M + Na]^+$.

1-[(1*R,2*R**)-1-(4-Chlorophenyl)-2,3-dimethylbutyl]-1,3-dimethyl-3-phenylurea (4e)**

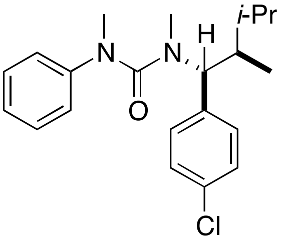

The compound was synthesised following the general procedure 1 starting from 108 mg (0.34 mmol) of urea ***E*-3e**. The desired product was obtained in 81% yield (100 mg) as a yellow oil.

***R*$_f$**: 0.5 (PE/EtOAc 8:2).

IR ν_{max} (film)/cm^{-1}: 2958, 1632 and 1594.

^{1}H NMR (300 MHz, $CDCl_3$): δ 7.30-7.15 (m, 6H, 6xAr*H*), 7.05 (tt, *J* = 8.7 and 1.2 Hz, 1H, Ar*H*), 6.87-6.83 (m, 2H, 2xAr*H*), 5.18 (d, *J* = 12.0 Hz, 1H, C*H*-N), 3.11 (s, 3H, N-CH_3), 2.23 (s, 3H, N-CH_3), 2.03 (m, 1H, CH-C*H*-CH_3), 1.71 (dsept, *J* = 2.4 and 6.9 Hz, 1H, C*H*-$(CH_3)_2$), 0.92 (d, *J* = 6.9 Hz, 6H, CH-$(CH_3)_2$) and 0.56 (d, *J* = 6.6 Hz, 3H, CH_3-CH).

^{13}C NMR (75 MHz, $CDCl_3$): δ 162.2 (C=O), 147.1 (C_{ar}), 137.8 (C_{ar}-Cl), 132.9 (C_{ar}), 130.1 (2xCH_{ar}), 129.3 (2xCH_{ar}), 128.3 (2xCH_{ar}), 124.7 (CH_{ar}), 124.3 (2xCH_{ar}), 61.4 (CH-N), 40.5 (N-CH_3), 37.1 (*C*H-CH_3), 30.6 (N-CH_3), 26.6 (*C*H-$(CH_3)_2$), 21.7 (CH-$(CH_3)_2$), 15.2 (CH-$(CH_3)_2$) and 10.4 (CH_3-CH).

HRMS–ES: *m/z* calcd for $C_{21}H_{28}N_2OCl$ 359.1885 found 359.1899 $[M + H]^+$.

1-[(1*R,2*S**)-1-(4-Chlorophenyl)-2,3-dimethylbutyl]-1,3-dimethyl-3-phenylurea (*epi*-4e)**

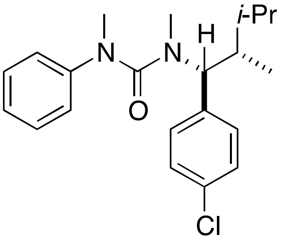

The compound was synthesised following the general procedure 1 starting from 100 mg (0.32 mmol) of urea ***Z*-3e**. The desired product was obtained in 80% yield (90 mg) as a yellow oil.

***R*$_f$**: 0.5 (PE/EtOAc 8:2).

IR ν_{max} (film)/cm^{-1}: 2958, 1637 and 1594.

^{1}H NMR (300 MHz, $CDCl_3$): δ 7.29-7.18 (m, 6H, 6x Ar*H*), 7.06 (tt, *J* = 7.5 and 1.2 Hz, 1H, Ar*H*), 6.92-6.88 (m, 2H, 2xAr*H*), 5.19 (d, *J* = 12 Hz, 1H, C*H*-N), 3.14 (s, 3H, N-CH_3), 2.26 (s, 3H, N-CH_3), 2.05 (m, 1H, C*H*-CH_3), 1.42 (dsept, *J* = 2.4 and 6.9 Hz, 1H, C*H*-$(CH_3)_2$), 0.85 (d, *J* = 6.9 Hz, 3H, CH-$(CH_3)_2$), 0.80 (d, *J* = 6.9 Hz, 3H, CH_3-CH) and 0.68 (d, *J* = 6.9 Hz, 3H, CH-$(CH_3)_2$).

^{13}C NMR (75 MHz, $CDCl_3$): δ 162.5 (C=O), 147.0 (C_{ar}), 137.2 (C_{ar}-Cl), 133.0 (C_{ar}), 130.0 (2xCH_{ar}), 129.3 (2xCH_{ar}), 128.4 (2xCH_{ar}), 124.6 (CH_{ar}), 124.4 (2xCH_{ar}), 61.8 (CH-N), 40.4 (N-CH_3), 37.3 (*C*H-CH_3), 30.5 (N-CH_3), 27.3 (*C*H-$(CH_3)_2$), 21.9 (CH-$(CH_3)_2$), 14.7 (CH-$(CH_3)_2$) and 9.6 (CH_3-CH).

HRMS–ES: *m/z* calcd for $C_{21}H_{27}N_2OClNa$ 381.1704 found 381.1692 $[M + Na]^+$.

1-[(1*R,2*R**)-2,3-Dimethyl-1-phenylbutyl]-3-(4-methoxyphenyl)-1,3-dimethylurea (4g)**

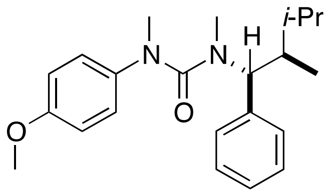

The compound was synthesised following the general procedure 1 in toluene for 1.5 h starting from 47 mg (0.15 mmol) of urea ***E*-3f**. The desired product was obtained in 85% yield (46 mg) as a yellow oil.

***R*f**: 0.5 (PE/EtOAc 8:2).

IR ν$_{max}$ (film)/cm^{-1}: 2960, 1641 and 1511.

^{1}H NMR (500 MHz, $CDCl_3$): δ 7.36 (d, *J* = 8.0 Hz, 2H, 2xAr*H*), 7.31 (t, *J* = 7.0 Hz, 2H, 2xAr*H*), 7.28-7.24 (m, 1H, Ar*H*), 6.80 (dt, *J* = 9.0 and 3.0 Hz, 2H, 2xAr*H*), 6.72 (dt, *J* = 9.0 and 3.0 Hz, 2H, 2xAr*H*), 5.23 (d, *J* = 11.5 Hz, 1H, C*H*-N), 3.77 (s, 3H, O-CH_3), 3.09 (s, 3H, N-CH_3), 2.26 (s, 3H, N-CH_3), 2.10 (m, 1H, C*H*-CH_3), 1.74 (dsept, *J* = 2.5 and 7.0 Hz, 1H, C*H*-$(CH_3)_2$), 0.94 (d, *J* = 7.0 Hz, 3H, $(CH_3)_2$-CH), 0.93 (d, *J* = 7.0 Hz, 3H, $(CH_3)_2$-CH) and 0.61 (d, *J* = 7.0 Hz, 3H, CH_3-CH).

^{13}C NMR (125 MHz, $CDCl_3$): δ 162.5 (C=O), 158.7 (C_{ar}-OCH_3), 140.3 (C_{ar}), 139.3 (C_{ar}), 128.8 (2xCH_{ar}), 128.1 (2xCH_{ar}), 127.1 (CH_{ar}), 126.1 (2xCH_{ar}), 114.4 (2xCH_{ar}), 62.0 (CH-N), 55.4 (O-CH_3), 41.0 (N-CH_3), 36.9 (*C*H-CH_3), 30.7 (N-CH_3), 26.6 (*C*H-$(CH_3)_2$), 21.8 (CH-$(CH_3)_2$), 15.3 (CH-$(CH_3)_2$) and 10.5 (CH_3-CH).

HRMS–ES: *m/z* calcd for $C_{22}H_{31}N_2O_2$ 355.2380 found 355.2388 $[M + H]^+$.

1-[(1R*,2S*)-2,3-Dimethyl-1-phenylbutyl]-3-(4-methoxyphenyl)-1,3-dimethylurea (epi-4g)

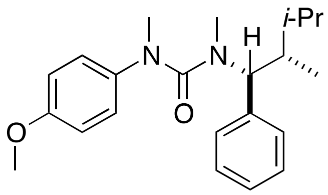

The compound was synthesised following the general procedure in toluene for 1.5 h starting from 40 mg (0.13 mmol) of urea ***Z*-3f**. The desired product was obtained in 85% yield (40 mg) as a yellow oil.

***R*f**: 0.5 (PE/EtOAc 8:2).

IR ν$_{max}$ (film)/cm^{-1}: 2960, 1643 and 1511.

^{1}H NMR (500 MHz, $CDCl_3$): δ 7.22-7.30 (m, 5H, 5xAr*H*), 6.81 (dt, *J* = 9.0 and 2.5 Hz, 2H, 2xAr*H*), 6.69 (dt, *J* = 9.0 and 2.5 Hz, 2H, 2xAr*H*), 5.22 (d, *J* = 12.0 Hz, 1H, C*H*-N), 3.73 (s, 3H, O-CH_3), 3.08 (s, 3H, N-CH_3), 2.24 (s, 3H, N-CH_3), 2.09 (m, 1H, CH_3-C*H*-CH), 1.46 (dsept, *J* = 2.5 and 7.0 Hz, 1H, C*H*-$(CH_3)_2$), 0.84 (d, *J* = 7.0 Hz, 3H, $(CH_3)_2$-CH), 0.78 (d, *J* = 7.5 Hz, 3H, CH_3-CH) and 0.68 (d, *J* = 7.0 Hz, 3H, $(CH_3)_2$-CH).

^{13}C NMR (125 MHz, $CDCl_3$): δ 162.8 (C=O), 156.7 (C_{ar}-OCH_3), 140.2 (C_{ar}), 138.7 (C_{ar}), 128.6 (2xCH_{ar}), 128.2 (2xCH_{ar}), 127.2 (CH_{ar}), 126.2 (2xCH_{ar}), 114.4 (2xCH_{ar}), 62.4 (CH-N), 55.4 (O-CH_3), 41.0 (N-CH_3), 37.0 (*C*H-CH_3), 30.5 (N-CH_3) 27.3 (*C*H-$(CH_3)_2$), 22.0 (CH-$(CH_3)_2$), 14.8 (CH-$(CH_3)_2$) and 9.7 (CH_3-CH).

HRMS–ES: *m/z* calcd for $C_{22}H_{31}N_2O_2$ 355.2380 found 355.2372 $[M + H]^+$.

1-[(1*R,2*R**)-1-(4-Chlorophenyl)-2,3-dimethyl-butyl]-3-(4-methoxyphenyl)-1,3-dimethylurea (4h)**

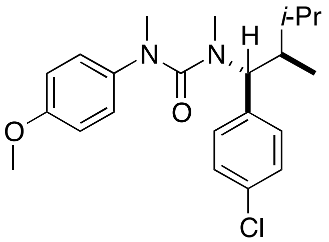

The product was prepared following the general procedure 1 starting from 40 mg of urea ***E*-3g**. The desired compound is obtained in 60% yield (30 mg) as an oil. Alternatively, the compound can be synthesised by treatment of the urea **6c** with sodium hydride (2 equiv), in dry THF at 0 °C for 30 min, followed by addition of methyl iodide (2 equiv), at r.t. for 24 h. The reaction was diluted with Et_2O and quenched with water. The mixture was extracted with EtOAc, dried ($MgSO_4$), and filtered, and the solvent was removed under reduced pressure. The desired compound was obtained in 60% yield after flash chromatography on silica gel (PE/EtOAc 9:1).

***R*$_f$**: 0.6 (PE/EtOAc 7:3)

IR ν_{max} (film)/cm^{-1}: 2958, 2361, 1634 and 1509.

^{1}H NMR (300 MHz, $CDCl_3$): δ 7.32-7.24 (m, 4H, 4xAr*H*), 6.88-6.73 (m, 4H, 4xAr*H*), 5.17 (d, *J* = 11.7 Hz, 1H, C*H*-N), 3.77 (s, 3H, O-C*H$_3$*), 3.07 (s, 3H, N-C*H$_3$*), 2.23 (s, 3H, N-C*H$_3$*), 2.03 (m, 1H, C*H*-CH_3), 1.71 (dsept, *J* = 6.9 and 2.4 Hz, 1H, C*H*-$(CH_3)_2$), 0.93 (d, *J* = 6.9 Hz, 3H, CH-(C*H$_3$*)$_2$), 0.92 (d, *J* = 6.9 Hz, 3H, CH-(C*H$_3$*)$_2$) and 0.58 (d, *J* = 6.9 Hz, 3H, C*H$_3$*-CH).

^{13}C NMR (75 MHz, $CDCl_3$): δ 162.5 (C=O), 156.9 (C_{ar}-OCH_3), 140.1 (C_{ar}), 138.0 (C_{ar}), 132.8 (C_{ar}-Cl), 130.1 (2xCH_{ar}), 128.3 (2xCH_{ar}), 126.2 (2xCH_{ar}), 114.5 (2xCH_{ar}), 61.4 (CH-N), 55.4 (O-CH_3), 41.1 (N-CH_3), 37.1 (*C*H-CH_3), 30.7 (N-CH_3), 26.6 (*C*H-$(CH_3)_2$), 21.8 (CH-(*C*H$_3$)$_2$), 15.1 (CH-(*C*H$_3$)$_2$) and 10.4 (CH_3-CH).

HRMS–ES: calcd for $C_{22}H_{29}ClN_2O_2$ 389.1990 found 389.1986 $[M + H]^+$.

3-(4-Methoxyphenyl)-1-methyl-1-[(*E*)-1-phenylprop-1-enyl]urea (E-5a)

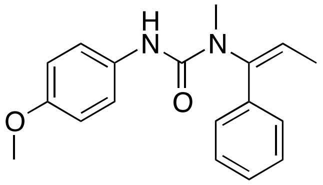

Propiophenone (1.00 g, 7.45 mmol, 1 equiv) was added to a solution of methylamine (8 M in EtOH, 4 equiv) in the presence of molecular sieves (1:1 w/w). The mixture was heated under microwave irradiation for 1 h at 125 °C (or alternatively at 50 °C for 48 h). The crude mixture was filtered through Celite and concentrated under vacuum. The obtained imine was solubilised in toluene (0.5 M) and treated with the 4-methoxyphenyl isocyanate (1 equiv), then stirred for 16 h at r.t. The solvent was evaporated and the crude was purified on silica gel (PE/EtOAc 8:2 + 1% NEt_3). The desired compound was obtained in 40% (0.88 g) as an oil.

***R*$_f$**: 0.2 (PE/EtOAC 8:2).

IR ν_{max} (film)/cm^{-1}: 3407, 3326, 2933, 2830, 1664, 1591 and 1508.

^{1}H NMR ($CDCl_3$, 300 MHz): δ 7.33-7.20 (m, 5H, 5xAr*H*), 7.12-7.09 (m, 2H, 2xAr*H*), 6.70-6.66 (m, 2H, 2xAr*H*), 6.47 (br. s, 1H, N*H*), 6.17 (q, *J* = 6.9 Hz, 1H, C*H*=C), 3.63 (d, *J* = 0.5 Hz, 3H, O-C*H$_3$*), 2.96 (s, 3H, N-C*H$_3$*) and 1.76 (d, *J* = 6.9 Hz, 3H, C*H$_3$*-CH).

^{13}C NMR ($CDCl_3$, 75 MHz): δ 155.6 (O-CH_3), 154.7 (C=O), 140.8 (C_{ar}), 135.8 (C_{ar}), 128.9 (2xCH_{ar}), 128.44 (CH_{ar}), 125.4 (2xCH_{ar}), 124.4 (*C*H=C), 121.47 (2xCH_{ar}), 121.40 (2xCH_{ar}), 114.0 (*C*=CH), 55.4 (O-CH_3), 34.0 (N-CH_3) and 13.6 (*C*H$_3$-CH). **LRMS** (ES): *m/z* 297 (100%, $M+H)^+$.

3-(4-Methoxyphenyl)-1-methyl-1-[(1*R**,2*R**)-2-methyl-1-phenylhexyl]urea (6a)

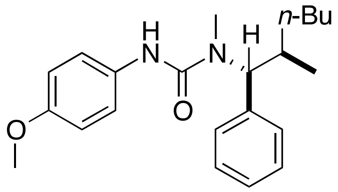

The compound was synthesised following the general procedure 2 starting from 53 mg (0.18 mmol) of urea **5a**. The desired product was obtained in 80% yield (40 mg) as a colourless oil.

***R*f**: 0.6 (PE/EtOAc 7:3).

IR ν_{max} (film)/cm^{-1}: 2956, 1633, 1600 and 1513.

^{1}H NMR (400 MHz, $CDCl_3$): δ 7.40-7.24 (m, 7H, 7xAr*H*), 6.85-6.81 (m, 2H, 2xAr*H*), 6.17 (br s, 1H, N*H*), 5.27 (d, *J* = 11.2 Hz, 1H, C*H*-N), 3.77 (s, 3H, O-CH_3), 2.76 (s, 3H, N-CH_3), 2.20 (m, 1H, C*H*-CH_3), 1.53-1.21 (m, 6H, CH_2-CH_2-CH_2), 0.93 (t, *J* = 6.8 Hz, 3H, CH_3-CH_2) and 0.84 (d, *J* = 6.4 Hz, 3H, CH_3-CH).

^{13}C NMR (100 MHz, $CDCl_3$): δ 156.0 (C=O), 155.7 (C_{ar}-OCH_3), 139.7 (C_{ar}), 132.2 (C_{ar}), 128.5 (2xCH_{ar}), 128.4 (2xCH_{ar}), 127.3 (CH_{ar}), 122.1 (2xCH_{ar}), 114.0 (2xCH_{ar}), 62.4 (CH-N), 55.5 (O-CH_3), 32.6 (CH_2-CH_2-CH_2), 32.2 (*C*H-CH_3), 29.0 (CH_2-CH_2-CH_3), 28.7 (N-CH_3), 23.0 (CH_2-CH_2), 17.1 (CH_3-CH) and 14.2 (CH_3-CH_2).

HRMS–ES: calcd 377.2199 for $C_{22}H_{30}N_2O_2Na$ found 377.2207 $[M + Na]^+$.

1-[(1*R**,2*R**)-1-(4-Chlorophenyl)-2-methylhexyl]-3-(4-methoxyphenyl)-1-methyl-urea (6b)

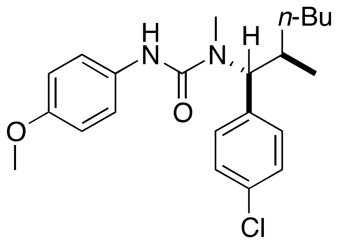

The compound was synthesised following the general procedure 2 starting from 98 mg (0.30 mmol) of urea **5b**. The desired product was obtained in 87% yield (100 mg) as a colourless oil.

***R*f**: 0.6 (PE/EtOAc 7:3).

IR ν_{max} (film)/cm^{-1}: 3325, 2956 and 1684.

^{1}H NMR (400 MHz, $CDCl_3$): δ 7.29-7.22 (m, 6H, 6xAr*H*), 6.77 (d, *J* = 8.8, 2H, 2xAr*H*), 6.26 (s, 1H, N*H*), 5.22 (d, *J* = 8.8Hz, 1H, C*H*-N), 3.73 (s, 3H, O-CH_3), 2.68 (s, 3H, N-CH_3), 2.12 (m, 1H, C*H*-CH_3), 1.46-1.12 (m, 6H, CH_2-CH_2-CH_2), 0.89 (t, *J* = 6.8 Hz, 3H, CH_3-CH_2) and 0.78 (d, *J* = 6.4 Hz, 3H, CH_3-CH).

^{13}C NMR (100 MHz, $CDCl_3$): δ 156.0 (C=O), 155.7 (C_{ar}-OCH_3), 138.2 (C_{ar}), 132.9 (C_{ar}), 132.0 (C_{ar}-Cl), 129.8 (2xCH_{ar}), 128.5 (2xCH_{ar}), 122.3 (2xCH_{ar}), 113.9 (2xCH_{ar}), 61.5 (CH-N), 55.4 (O-CH_3), 32.4 (CH_2-CH), 32.0 (*C*H-CH_3), 28.8 (CH_2-CH_2-CH_2), 28.6 (N-CH_3), 22.9 (CH_2-CH_3), 16.9 (CH_3-CH) and 14.1 (CH_3-CH_2).

HRMS–ES: calcd 387.1844 for $C_{22}H_{28}N_2O_2Cl$ found 387.1847 $[M - H]^-$.

1-[(1*R,2*R**)-1-(4-Chlorophenyl)-2,3-dimethylbutyl]-3-(4-methoxyphenyl)-1-methylurea (6c)**

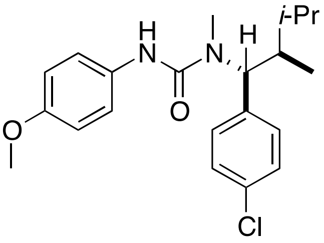

The compound was synthesised following the general procedure 2 starting from 99 mg (0.30 mmol) of urea **5b**. The desired product was obtained in 98% yield (110 mg) as white solid. The compound was recrystallized from petroleum ether.

***R*f**: 0.6 (PE/EtOAc 7:3).

mp: 157–158 °C (PE).

IR ν_{max} (film)/cm^{-1}: 3361, 1643 and 1511.

^{1}H NMR (400 MHz, $CDCl_3$): δ 7.36-7.30 (m, 4H, 4xAr*H*), 7.28-7.24 (m, 2H, 2xAr*H*), 6.84-6.80 (m, 2H, 2xAr*H*), 5.40 (d, *J* = 12.0 Hz, 1H, C*H*-N), 3.77 (s, 3H, O-C*H*$_3$), 2.73 (s, 3H, N-C*H*$_3$), 2.15 (m, 1H, C*H*-CH_3), 1.82 (dsept, *J* = 6.8 and 2.4 Hz, 1H, C*H*-$(CH_3)_2$), 1.01 (d, *J* = 6.8 Hz, 3H, CH-(C*H*$_3$)$_2$), 0.88 (d, *J* = 6.8 Hz, 3H, CH-(C*H*$_3$)$_2$) and 0.67 (d, *J* = 6.8 Hz, 3H C*H*$_3$-CH).

^{13}C NMR (100 MHz, $CDCl_3$): δ 155.7 (C=O), 155.7 (*C*$_{ar}$-OCH_3), 138.3 (C_{ar}), 133.0 (C_{ar}), 132.0 (C_{ar}-Cl), 129.8 (2xCH_{ar}), 128.6 (2xCH_{ar}), 122.3 (2xCH_{ar}), 114.0 (2xCH_{ar}), 59.4 (CH-N), 55.5 (O-CH_3), 37.0 (*C*H-CH_3), 28.6 (N-CH_3), 27.1 (*C*H-$(CH_3)_2$), 21.8 (CH-(*C*H$_3$)$_2$), 15.1 (CH-(*C*H$_3$)$_2$) and 10.5 (*C*H$_3$-CH).

HRMS–ES: calcd 373.1688 for $C_{21}H_{26}N_2O_2Cl$ found 373.1689 (M-H)$^-$.

Elementary Anal. calcd for C 67.28, H 7.26 and N 7.47 found C 67.56, H 7.52 and N 7.49.

1-[(1*R,2*R**)-1-(4-Chlorophenyl)-2,3,3-trimethylbutyl]-3-(4-methoxyphenyl)-1-methylurea (6d)**

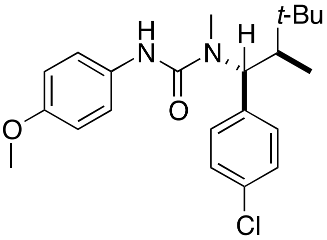

The compound was synthesised following the general procedure 2 starting from 98 mg (0.29 mmol) of urea **5b**. The desired product was obtained in 98% yield (120 mg) as a clear yellow oil.

***R*f**: 0.6 (PE/EtOAc 7:3).

IR ν_{max} (film)/cm^{-1}: 3341, 2953, 2834, 1664, 1534 and 1514.

^{1}H NMR (400 MHz, $CDCl_3$): δ 7.27-7.22 (m, 6H, 6xAr*H*), 6.80-6.76 (m, 2H, 2xAr*H*), 6.28 (br s, 1H, N*H*), 5.56 (d, *J* = 10.8 Hz, 1H, C*H*-N), 3.74 (s, 3H, O-C*H*$_3$), 2.68 (s, 3H, N-C*H*$_3$), 2.06 (dq, *J* = 10.8 and 6.8 Hz, 1H, C*H*-CH_3), 1.02 (s, 9H, 3x(C*H*$_3$)-C) and 0.69 (d, *J* = 6.8 Hz, 3H, C*H*$_3$-CH).

^{13}C NMR (100 MHz, $CDCl_3$): δ 155.6 (C=O), 155.3 (C_{ar}-OCH_3), 139.5 (C_{ar}), 132.4 (C_{ar}), 131.9 (C_{ar}-Cl), 129.5 (2xCH_{ar}), 128.4 (2xCH_{ar}), 122.4 (2xCH_{ar}), 113.8 (2xCH_{ar}), 59.1 (CH-N), 55.4 (O-CH_3), 40.5 (*C*H-CH_3), 33.5 (*C*-$(CH_3)_3$), 30.0 (N-CH_3), 28.3 (3xC-*C*H$_3$) and 14.8 (*C*H$_3$-CH).

HRMS–ES: calcd 387.1844 for $C_{22}H_{28}N_2O_2Cl$ found 387.1844 (M-H)$^-$.

tert-Butyl _N_-methyl-_N_-(1-phenylvinyl)carbamate (9)

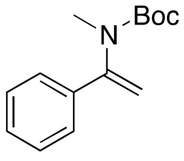

Acetophenone (2.00 g, 16.6 mmol, 1 equiv) was treated with a solution of methylamine (8 M in EtOH, 4 equiv) in the presence of M.S. 4Å. The reaction was stirred for 48 h at room temperature (alternatively 0.5 h in a microwave at 125 °C). The crude is filtered through Celite. The solvent was removed under vacuum. The crude was solubilised in toluene and Boc_2O was added (1 equiv) to the reaction mixture. The reaction mixture was stirred for 16 h under reflux. The crude was washed with H_2O, the organic phases were combined and dried with $MgSO_4$, and solvent was removed under reduced pressure. The desired compound was obtained without further purification in 80% yield (3.10 g) as an oil.

***R*f**: 0.5 (PE/EtOAc 8:2).

IR ν$_{max}$ (film)/cm^{-1}: 2976, 1807, 1755, 1697 and 1625.

^{1}H NMR (300 MHz, $CDCl_3$): δ 7.38-7.26 (m, 5H, 5xAr*H*), 5.27 (s, 1H, C=C*H*$_2$), 5.05 (s, 1H, C=C*H*$_2$), 3.22 (s, 3H, N-C*H*$_3$) and 1.20 (s, 9H, C-(C*H*$_3$)$_3$).

^{13}C NMR (75 MHz, $CDCl_3$): δ 154.6 (C=O), 149.3 (*C*=CH$_2$), 138.9 (C$_{ar}$), 128.2 (2xCH$_{ar}$), 127.9 (CH$_{ar}$), 125.5 (2xCH$_{ar}$), 107.9 (C=*C*H$_2$), 80.0 (*C*-(CH$_3$)$_3$), 37.4 (N-CH$_3$) and 27.9 (3x(*C*H$_3$)-C).

HRMS–ES: calcd for $C_{14}H_{19}NO_2Na$ 256.1305 found 256.1308 $[M + Na]^+$.

tert-Butyl *N*-methyl-*N*-[(*E*)-1-phenylprop-1-enyl]carbamate (*E*-10)

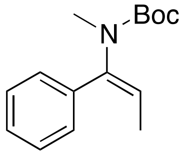

Propiophenone (2.00 g, 14.9 mmol, 1 equiv) was treated with a solution of methylamine (8 M in EtOH, 4 equiv) in the presence of M.S. 4Å. The reaction was stirred for 1 h under microwave irradiation (125 °C). The crude was filtered through Celite. The solvent was removed under vacuum and the crude solubilised in toluene. Boc_2O (1 equiv) was added to the reaction mixture, and the reaction mixture was stirred for 16 h under reflux. The crude was washed with H_2O, the organic phases were combined and dried ($MgSO_4$), and the solvent was removed under reduce pressure. The desired compound was obtained after distillation under reduced pressure in 70% yield (2.58 g) as a white solid. The stereochemistry is confirmed by nOe experiments and X-ray structure.

mp: 20 °C (PE).

***R*f**: 0.5 (PE/EtOAc 8:2).

IR ν$_{max}$ (film)/cm^{-1}: 2977 and 1686.

^{1}H NMR (500 MHz, $CDCl_3$): δ 7.33-7.23 (m, 5H, 5xAr*H*), 5.65 (q, *J* = 7.0 Hz, 1H, C=C*H*), 3.12 (s, 3H, N-C*H*$_3$), 1.77 (d, *J* = 7.0 Hz, 3H, C*H*$_3$-CH) and 1.22 (br s, 9H, 3xC-C*H*$_3$).

^{13}C NMR (125 MHz, $CDCl_3$): δ 155.2 (C=O), 141.5 (*C*=CH), 128.4 (2xCH$_{ar}$), 127.9 (2xCH$_{ar}$), 127.3 (CH$_{ar}$), 120.7 (C=*C*H), 79.7 (*C*-(CH$_3$)$_3$), 37.6 (N-CH$_3$), 28.1 (3x(*C*H$_3$)-C) and 14.0 (*C*H$_3$-CH).

HRMS–ES: calcd for $C_{15}H_{21}NO_2Na$ 270.1465 found 270.1468 $[M + Na]^+$.

***tert*-Butyl *N*-methyl-*N*-[(*Z*)-1-phenylprop-1-enyl]carbamate (*Z*-10)**

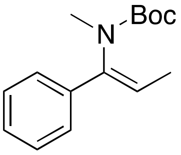

Carbamate **(*E*)-10** (100 mg, 0.40 mmol) was solubilised in dry THF and cooled to −40 °C. LDA (2 equiv) was added slowly and the reaction mixture was stirred for 2 h at this temperature. The reaction was quenched slowly with methanol, and the mixture was washed with NH_4Cl and extracted with EtOAc. The organic phase was dried ($MgSO_4$), filtered and concentrated under reduced pressure. The crude was purified by flash chromatography, and the desired compound was obtained in 90% yield (colourless oil).

***R*$_f$**: 0.5 (PE/EtOAc 8:2)

IR ν$_{max}$ (film)/cm^{-1}: 2975, 1703 and 1699.

^{1}H NMR (400 MHz, $CDCl_3$): δ 7.30-7.19 (m, 5H, 5xAr*H*), 5.84 (q, *J* = 7.2 Hz, 1H, C=C*H*-CH_3), 2.97 (s, 3H, N-C*H*$_3$), 1.71 (d, *J* = 7.2 Hz, 3H, C*H*$_3$-CH) and 1.27 (br s, 9H, 3x(C*H*$_3$)-C).

^{13}C NMR (100 MHz, $CDCl_3$): δ 155.3 (C=O), 141.1 (*C*=CH), 138.1 (C$_{ar}$), 128.3 (2xCH$_{ar}$), 127.3 (CH$_{ar}$), 125.2 (2xCH$_{ar}$), 120.9 (C=*C*H), 79.5 (*C*-(CH_3)$_3$), 35.2 (N-CH_3), 28.2 (3x(*C*H$_3$)-C) and 13.6 (*C*H$_3$-CH).

HRMS–ES: calcd 270.1465 for $C_{15}H_{21}NO_2Na$ (M+Na$^+$) found 270.1473.

[(1*R*,2*R*,5*S*)-2-Isopropyl-5-methyl-cyclohexyl] *N*-methyl-*N*-(1-phenylvinyl)carbamate (11)

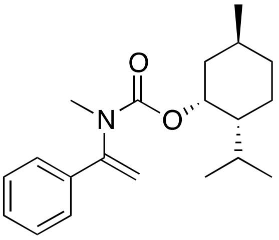

Acetophenone (2.00 g, 16.6 mmol, 1 equiv) was treated with a solution of methylamine (8 M in EtOH, 4 equiv) in the presence of M.S. 4Å. The reaction was stirred for 0.5 h under microwave irradiation at 125 °C. The crude was filtered through Celite. The solvent was removed under vacuum. The crude was solubilised in toluene, and menthylchloroformate was added (1 equiv) to the reaction mixture. The reaction was stirred for 16 h under reflux. The crude was washed with H_2O, the organic phases were combined and dried with $MgSO_4$, and solvent was removed under reduced pressure. The desired compound was obtained as a white solid without further purification in 70% yield (3.8 g).

***R*$_f$**: 0.5 (PE/EtOAc 8:2).

IR ν$_{max}$ (powder)/cm^{-1}: 2954, 2867, 2247, 1688 and 1628.

^{1}H NMR (300 MHz, $CDCl_3$): δ 7.38-7.28 (m, 5H, 5xAr*H*), 5.40 (s, 1H, C=C*H*$_2$), 5.12 (s, 1H, C=C*H*$_2$), 4.44 (dt, *J* = 4.5 and 10.8 Hz, 1H, C*H*-O), 3.22 (s, 3H, N-C*H*$_3$), 2.01-1.94 (m, 1H, C*H*$_2$-CH-O), 1.63-1.51 (m, 2H, C*H*$_2$-CH-CH-O and C*H*$_2$-CH-CH_3), 1.47-1.34 (m, 2H, C*H*-(CH_3)$_2$ and C*H*-CH_3), 1.11-1.04 (m, 1H, C*H*-CH-(CH_3)$_2$), 0.94 (dq, *J* = 4.2 and 12.6 Hz, 1H, C*H*$_2$-CH-CH-O), 0.84 (d, *J* = 6.6 Hz, 3H, CH-(C*H*$_3$)$_2$), 0.80-0.73 (m, 1H, C*H*$_2$-CH-CH_3), 0.69 (br d, *J* = 6.9 Hz, 4H, (C*H*$_3$-CH) and C*H*$_2$-CH-O) and 0.65 (d, *J* = 6.9 Hz, 3H, (CH-C*H*$_3$)$_2$).

^{13}C NMR (75 MHz, $CDCl_3$): δ 155.5 (C=O), 148.6 (*C*=CH_2), 138.0 (C$_{ar}$), 128.4 (2xCH$_{ar}$), 128.2 (CH$_{ar}$), 125.5 (2xCH$_{ar}$), 109.6 (C=CH_2), 75.6 (CH-O), 49.2 (*C*H-CH-(CH_3)$_2$), 41.0 (*C*H$_2$-CH-O), 37.6 (N-CH_3), 34.2 (*C*H$_2$-CH-CH_3), 31.3 (*C*H-(CH_3)$_2$), 25.7 (*C*H-CH_3), 23.2 (*C*H$_2$-CH-CH-O), 22.0 (CH-(*C*H$_3$)$_2$), 20.8 (*C*H$_3$-CH) and 16.1 (CH-(*C*H$_3$)$_2$). **HRMS–ES**: calcd for $C_{20}H_{29}N_2ONa$ 316.2271 found 316.2271[M + Na]$^+$. **Elementary Anal.** calcd for C 76.15, H 9.27, N 4.44 found: C 76.14, H 9.24, N 4.42.

tert-Butyl *N*-methyl-*N*-(1-phenylpentyl)carbamate (12a)

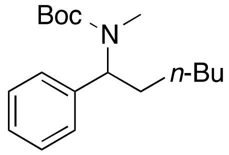

The compound was synthesised following the general procedure 3 starting from 50 mg (0.21 mmol) of vinyl carbamate **9**. The desired product was obtained in 61% yield (40 mg) as a colourless oil. The NMR analysis shows the presence of two rotamers.

$\boldsymbol{R_f}$: 0.7 (PE/EtOAc 8:2).

IR ν_{max} (film)/cm^{-1}: 3361, 2929 and 1676.

^{1}H NMR (300 MHz, $CDCl_3$): δ 7.36-7.21 (m, 10H, 10xAr*H* from both rotamers), 5.37 (br s, 1H, C*H*-N from 1st rotamer), 5.23 (br s, 1H, C*H*-N from 2nd rotamer), 2.55 (br s, 6H, N-CH_3 from both rotamers), 1.87 (br s, 4H, CH_2-CH from both rotamers), 1.49 (s, 18H, 3x(CH_3)-C from both rotamers), 1.37-1.34(m, 12H, CH_2-CH_2-CH_2 from both rotamers) and 0.90 (br t, *J* = 6.6 Hz, 6H, CH_3-CH_2 from both rotamers).

^{13}C NMR (75 MHz, $CDCl_3$): δ 156.2 (C=O), 140.9 (C_{ar}), 128.3 (2xCH_{ar}), 127.4 (CH_{ar}), 127.0 (2xCH_{ar}), 79.4 (*C*-(CH_3)$_3$), 57.0 (CH-N), 31.6 (CH-CH_2-*C*H_2), 30.0 (*C*H_2-CH), 28.5 (3x(*C*H_3)-C), 28.0 (N-CH_3), 26.0 (CH_2-*C*H_2-CH_2), 22.6 (*C*H_2-CH_3) and 14.0 (*C*H_3-CH_2).

HRMS–ES: calcd for $C_{18}H_{29}NO_2Na$ 314.2091 found 314.2094 $[M + Na]^+$.

tert-Butyl *N*-methyl-*N*-(3-methyl-1-phenyl-butyl)carbamate (12b)

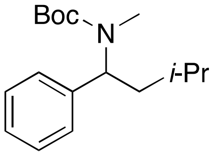

The compound was synthesised following the general procedure 3 starting from 40 mg (0.17 mmol) of carbamate **9**. The desired product was obtained in 61% yield (29 mg) as a colourless oil. The NMR analysis shows the presence of two rotamers.

$\boldsymbol{R_f}$: 0.7 (PE/EtOAc 8:2).

IR ν_{max} (film)/cm^{-1}: 2955 and 1686.

^{1}H NMR (300 MHz, $CDCl_3$): δ 7.35-7.23 (m, 10H, 10xAr*H* from both rotamers), 5.50 (br s, 1H, C*H*-N from 1st rotamer), 5.34 (br s, 1H, C*H*-N from 2nd rotamer), 2.55 (s, 6H, N-CH_3 from both rotamers), 1.83 (br s, 2H, C*H*-(CH_3)$_2$, from both rotamers), 1.62-1.60 (m, 4H, CH-CH_2, from both rotamers), 1.50 (s, 18H, 3x(CH_3)-C, from both rotamers), 1.00 (d, *J* = 6.3 Hz, 6H, CH_3-CH, from both rotamers) and 0.98 (d, *J* = 6.3 Hz, 6H, CH_3-CH, from both rotamers).

^{13}C NMR (75 MHz, $CDCl_3$): δ 156.1 (C=O), 141.0 (C_{ar}), 128.3 (2xCH_{ar}), 127.4 (CH_{ar}), 127.1 (2xCH_{ar}), 79.5 (*C*-(CH_3)$_3$), 55.5 (CH-N), 39.2 (CH-*C*H_2), 28.5 (N-CH_3), 28.0 (3x(*C*H_3)-C), 24.8 (*C*H-(CH_3)$_2$), 23.5 (CH-*C*H_3) and 21.9 (CH-*C*H_3).

HRMS–ES: calcd for $C_{17}H_{27}NO_2Na$ 300.1934 found 300.1936 $[M + Na]^+$.

tert-Butyl *N*-(3,3-dimethyl-1-phenyl-butyl)-*N*-methyl-carbamate (12c)

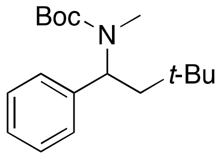

The compound was synthesised following the general procedure 3 starting from 50 mg (0.21 mmol) of carbamate **9**. The desired product was obtained in 80% yield (50 mg) as a colourless oil.

The NMR analysis shows the presence of two rotamers.

***R*$_f$**: 0.7 (PE/EtOAc 8:2).

IR ν_{max} (film)/cm^{-1}: 2955, 2357 and 1676.

^{1}H NMR (300 MHz, $CDCl_3$): δ 7.28-7.18 (m, 10H, 10xAr*H* from both rotamers), 5.57 (br s, 1H, C*H*-N from 1st rotamer), 5.41 (br s, 1H, C*H*-N from 2nd rotamer) 2.52 (s, 6H, N-CH_3, from both rotamers), 1.85 (dd, *J* = 14.4 and 9.9 Hz, 2H, CH-CH_2, from both rotamers), 1.71 (dd, *J* = 14.4 and 3.9 Hz, 2H, CH-CH_2, from both rotamers), 1.48 (br s, 18H, 3x(CH_3)-C, from both rotamers) and 0.94 (s, 18H, 3x(CH_3)-C, from both rotamers).

^{13}C NMR (75 MHz, $CDCl_3$): δ 155.3 (C=O), 142.1 (C_{ar}), 128.3 (2xCH_{ar}), 127.4 (2xCH_{ar}), 127.0 (CH_{ar}), 79.6 (*C*-($CH_3)_3$), 79.4 (O-*C*-($CH_3)_3$), 54.9 (CH-N from 2nd rotamer), 53.8 (CH-N from 1st rotamer), 42.8 (*C*H_2-CH), 30.6 (3xCH-C-*C*H_3), 29.7 (3xO-C-*C*H_3) and 28.6 (N-CH_3).

HRMS–ES: calcd for $C_{18}H_{29}NO_2Na$ 314.2091 found 314.2099 $[M + Na]^+$.

(1*R**,2*R**)-*N*,2-Dimethyl-1-phenylhexan-1-amine (13a)

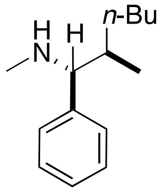

The compound was prepared following the general procedure 4 starting from 104 mg (0.42 mmol) of carbamate ***E*-10**. The desired product was obtained in 70% yield (60 mg) as an oil after flash chromatography (PE/DCM 1:1).

***R*$_f$**: 0.4 (PE/DCM 1:1).

IR ν_{max} (film)/cm^{-1}: 2975, 1703 and 1699.

^{1}H NMR (300 MHz, $CDCl_3$): δ 7.35-7.20 (m, 5H, 5xAr*H*), 3.32 (d, *J* = 6.6 Hz, 1H, C*H*-N), 2.25 (s, 3H, N-CH_3), 1.74 (m, 1H, C*H*-CH_3), 1.52 (m, 1H, CH-CH_2), 1.29 (m, 5H, CH_2-CH_2-CH_3 and N*H*), 1.06 (m, 1H, CH-CH_2), 0.90 (t, *J* = 6.9 Hz, 3H, CH_3-CH_2) and 0.74 (d, *J* = 6.6 Hz, 3H, CH_3-CH).

^{13}C NMR (75 MHz, $CDCl_3$): δ 142.3 (C_{ar}), 128.1 (2xCH_{ar}), 127.9 (2xCH_{ar}), 126.6 (CH_{ar}), 70.4 (CH-N), 39.0 (*C*H-CH_3), 34.9 (N-CH_3), 33.3 (*C*H_2-CH), 29.4 (*C*H_2-CH_2-CH_3), 23.0 (*C*H_2-CH_3), 16.0 (*C*H_3-CH) and 14.1 (*C*H_3-CH_2).

HRMS–ES: calcd for $C_{14}H_{24}N$ 206.1904 found 206.1902 $[M + H]^+$.

(1*R,2*R**)-*N*,2,3-Trimethyl-1-phenylbutan-1-amine (13b)**

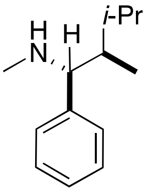

The compound was prepared following the general procedure 4 starting from 97 mg (0.39 mmol) of carbamate ***E*-10** The desired product was obtained in 80% yield (60 mg) as an oil.

***R*$_f$**: 0.4 (PE/DCM 1:1).

IR ν_{max} (film)/cm^{-1}: 2960, 2872 and 1682.

^{1}H NMR (300 MHz, $CDCl_3$): δ 7.35-7.22 (m, 5H, 5xAr*H*), 3.34 (d, *J* = 9 Hz, 1H, C*H*-N), 2.95 (br s, 1H, N*H*), 2.21 (s, 3H, N-C*H$_3$*), 2.04 (m, 1H, C*H*-(CH_3)$_2$), 1.68 (m, 1H, C*H*-CH_3), 0.97 (d, *J* = 6.6 Hz, 3H, CH-(C*H$_3$*)$_2$), 0.82 (d, *J* = 6.9 Hz, 3H, CH-(C*H$_3$*)$_2$) and 0.53 (d, *J* = 6.9 Hz, 3H, CH-C*H$_3$*).

^{13}C NMR (75 MHz, $CDCl_3$): δ 142.2 (C_{ar}), 128.2 (2xCH_{ar}), 128.1 (2xCH_{ar}), 127.0 (CH_{ar}), 68.3 (CH-N), 44.1 (*C*H-CH_3), 34.3 (N-CH_3), 27.6 (*C*H-(CH_3)$_2$), 21.8 (CH-(*C*H$_3$)$_2$), 16.0 (CH-(*C*H$_3$)$_2$) and 10.8 (CH-*C*H$_3$).

HRMS–ES: calcd for $C_{13}H_{22}N$ 192.1747 found 192.1740 $[M + H]^+$.

(1*R,2*S**)-*N*,2,3-Trimethyl-1-phenylbutan-1-amine (*epi*-13b)**

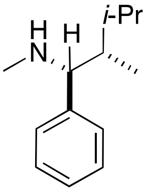

The carbamate ***Z*-10** (98 mg, 0.40 mmol) was solubilised in dry toluene and the mixture was cooled to −40 °C. Isopropyllithium was added slowly and the reaction was stirred for 24 h at −40 °C. The reaction was quenched by slow addition of methanol. The solvent was removed under reduced pressure and the crude solubilised in TFA. The reaction was stirred for 1 h at r.t. The reaction mixture was diluted in DCM and washed with $NaHCO_3$ (1 M). The organic phase was dried ($MgSO_4$) and filtered, and the solvent was removed under reduced pressure. The desired compound was obtained in 50% yield (40 mg) as an oil.

8:2 mixture of 2 diastereomers.

Only the main diastereomer is described

***R*$_f$**: 0.4 (PE/DCM 1:1).

IR ν_{max} (film)/cm^{-1}: 3024, 2958, 2872 and 2790.

^{1}H NMR (300 MHz, $CDCl_3$): δ 7.35-7.22 (m, 5H, 5xAr*H*), 3.38 (d, *J* = 7.8 Hz, 1H, C*H*-N), 2.21 (s, 3H, N-C*H$_3$*), 1.56 (m, 1H, C*H*-(CH_3)$_2$), 1.44 (m, 1H, C*H*-CH_3), 0.92 (d, *J* = 6.9 Hz, 3H, CH-(C*H$_3$*)$_2$), 0.88 (d, *J* = 6.9 Hz, 3H, CH-(C*H$_3$*)$_2$), 0.77 (d, *J* = 6.9 Hz, 3H, CH-C*H$_3$*).

^{13}C NMR (75 MHz, $CDCl_3$): δ 143.6 (C_{ar}), 128.1 (2xCH_{ar}), 127.7 (2xCH_{ar}), 126.6 (CH_{ar}), 68.4 (CH-N), 45.3 *C*H-CH_3), 34.7 (N-CH_3), 28.7 (*C*H-(CH_3)$_2$), 21.8 (CH-(*C*H$_3$)$_2$), 17.1 (CH-*C*H$_3$) and 10.6 (CH-(*C*H$_3$)$_2$).

HRMS–ES: calcd for $C_{13}H_{22}N$ 192.1747 found 192.1743 $[M + H]^+$.

(1*R,2*R**)-*N*,2,3,3-Tetramethyl-1-phenylbutan-1-amine (13c)**

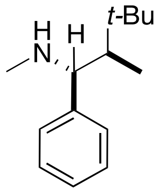

The compound was prepared following the general procedure 4 starting from 103 mg (0.42 mmol) of carbamate ***E*-10**. The desired product was obtained in 81% yield (70 mg) as an oil.

***R*f**: 0.4 (PE/DCM 1:1).

IR ν_{max} (film)/cm^{-1}: 2960, 2868 and 1676.

^{1}H NMR (300 MHz, $CDCl_3$): δ 7.28-7.03 (m, 5H, 5xAr*H*), 3.70 (d, *J* = 4.4 Hz, 1H, C*H*-N), 3.33 (br s, 1H, NH), 2.17 (s, 3H, N-C*H*$_3$), 1.64 (dq, *J* = 4.4 and 7.2 Hz, 1H, C*H*-CH_3), 0.81 (s, 9H (3x(C*H*$_3$)-C) and 0.79 (d, *J* = 7.2 Hz, 3H, CH-C*H*$_3$).

^{13}C NMR (75 MHz, $CDCl_3$): δ 141.3 (C_{ar}), 129.1 (2xCH_{ar}), 127.9 (2xCH_{ar}), 127.0 (CH_{ar}), 66.4 (CH-N), 48.5 (*C*H-CH_3), 34.1 (*C*-$(CH_3)_3$), 33.5 (N-CH_3), 28.3 (3x(*C*H$_3$)-C) and 11.3 (CH_3-CH).

HRMS–ES: calcd for $C_{14}H_{24}N$ 206.1903 found 206.1900 $[M + H]^+$.

[(2*R*,5*S*)-2-Isopropyl-5-methyl-cyclohexyl]*N*-methyl-*N*-(3-methyl-1-phenylbutyl)carbamate (14)

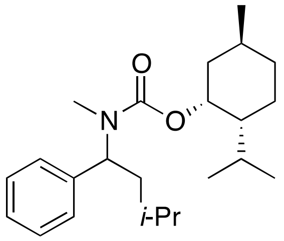

The desired carbamate was synthesised following the general procedure 4 starting from 52 mg (0.16 mmol) of carbamate **11** in 60% yield as an oil.

The presence of two diastereomers makes the assignment impossible.

***R*f**: 0.5 (PE/EtOAc 7:3).

^{1}H NMR (400 MHz, DMSO-d_6, 383.1 K): δ 7.25-7.13 (m, 10H, 10xAr*H* for both isomers), 5.21 (t, *J* = 5.6 Hz, 1H, C*H*-N one isomer), 5.18 (t, *J* = 6.0 Hz, 1H, C*H*-N one isomer), 4.46 (dt, *J* = 4.4 and 10.8 Hz, 2H, C*H*-O both isomer), 2.81 (s, 6H, N-CH_3 both isomers), 1.88-1.71 (m, 6H), 1.65-1.25 (m, 14H), 1.25-1.03 (m, 4H), 0.87 (d, *J* = 6.8 Hz, 12H, 2xCH-(C*H*$_3$)$_2$ both isomers), 0.81-0.76 (m, 12H, 2xCH-(C*H*$_3$)$_2$ both isomers) and 0.67 (d, *J* = 6.8 Hz, 6H, 3xC*H*$_3$-CH both isomers).

^{13}C NMR (100 MHz, DMSO-d_6, 383.1 K): δ 155.2, 140.3, 140.2, 127.7, 127.7, 126.5, 126.4, 126.4, 74.1, 55.4, 55.3, 46.7, 40.8, 40.8, 39.5, 38.7, 38.6, 33.5, 33.4, 30.4, 27.9, 27.8, 25.8, 25.7, 24.3, 24.2, 23.2, 23.1, 22.3, 22.1, 21.4, 21.2, 21.1, 19.88, 19.8, 16.0 and 15.9.

References

1. Lefranc J.; Tetlow D. J.; Donnard M.; Minassi A.; Gálvez E.; Clayden J. *Org. Lett.* **2011**, *13*, 296-299.

2. Clayden J.; Donnard M.; Lefranc J.; Minassi A.; Tetlow D. J. *J. Am. Chem. Soc.* **2010**, *132*, 6624-6625.
